# Selenium in Cancer Rehabilitation—A Retrospective Study from a Specialized Clinic

**DOI:** 10.3390/nu15173827

**Published:** 2023-09-01

**Authors:** Christina Pfister, Joerg Schoenemann

**Affiliations:** 1 biosyn Arzneimittel GmbH, Schorndorfer Straße 32, 70734 Fellbach, Germany; 2 Inselsberg Klinik Wicker GmbH & Co. OHG, Fischbacher Straße 36, 99891 Bad Tabarz, Germany

**Keywords:** selenium, zinc, micronutrient dietary intake, cancer, rehabilitation, health-related quality of life (HRQOL)

## Abstract

Background: Micronutrient deficiencies are common at the time of cancer diagnosis and are associated with worse prognosis. Little is known about them in cancer rehabilitation. Methods: Data from routine health-related quality of life (HRQOL) were analyzed at an inpatient cancer rehabilitation center. Rehabilitation patients completed the EORTC QLQ-C30 questionnaire before and after multidisciplinary rehabilitation treatment and three months after discharge. Selenium and zinc status were measured in whole blood at these three time points. In case of selenium deficiency, up to 600 µg selenium per day as sodium selenite was supplemented for three weeks during and for three months after rehabilitation. Results: A total of 271 patients (breast, colon, and pancreatic cancer) were included in the analysis. There was clinically meaningful improvement in many domains of the EORTC QLQ-C30 during rehabilitation. However, the effect often waned in the three months after. Prevalence for selenium deficiency varied between 34 to 90% depending on cancer type (breast < colon < pancreas). In contrast, zinc deficiency was rare. Daily selenium supplementation of 600 µg was more efficient to correct selenium deficiency compared to 300 µg selenium per day. Rehabilitation and increasing selenium status after rehabilitation were associated with improved global quality of life, physical and emotional functioning, and fatigue. In cancer patients with decreasing selenium status, values of global quality of life, physical and emotional functioning, and fatigue were back to the values at the beginning of rehabilitation. Conclusions: Selenium deficiency is common in cancer patients admitted to a cancer rehabilitation clinic. Selenium supplementation during rehabilitation effectively corrected selenium deficiency in most cases. The positive effects of rehabilitation persisted longer when selenium status did not decrease after rehabilitation.

## 1. Introduction

Rehabilitation is defined as “a process to restore mental and/or physical abilities lost to injury or disease, in order to function in a normal or near-normal way” [1]. As cancer has increasingly become a chronic disease, cancer patients can benefit from rehabilitation before, during, and after treatment [1]. A recent systematic review showed that 71% of the included trials reported statistically significant results after cancer rehabilitation for at least one functional outcome [2].

Many patients experience unwanted weight loss even before cancer diagnosis, which indicates malnutrition, as weight loss is its hallmark [3]. Side effects of cancer therapy, such as emesis, nausea, pain, and gastrointestinal disorders, can lead to a reduced food intake [3]. Also, metabolic changes contribute to cancer-associated malnutrition [4]. Malnutrition at the time of diagnosis affects survival [5]. A weight loss of 5–10% during radiotherapy had a significant negative impact on 5-year overall survival and disease-specific survival [5].

Pressoir et al. showed that one in three cancer patients are malnourished [6]. Prevalence of malnutrition was lowest in breast cancer patients, followed by patients with colon cancer [6]. In upper digestive cancer, such as pancreatic cancer, prevalence of malnutrition was highest.

Malnutrition is characterized by insufficient energy and macronutrient intake [3]. However, reduced food intake can also lead to micronutrient deficiencies—so-called hidden hunger [7]. In addition, micronutrient deficiencies can also occur without a deficit in energy intake [7]. 

Selenium is an essential trace element associated with the incidence of certain types of cancer [8,9]. Insufficient selenium intake has been estimated to affect up to one billion people worldwide [10]. Selenium deficiency increases the risk for thyroid and cardiovascular diseases [11,12,13,14,15]. Selenium plays an important role in antioxidant defense [16,17], and its deficiency impairs the immune system, increasing the risk for infections [18]. 

As fellow trace element and antioxidant, zinc is part of over 300 enzymes [19]. Zinc deficiency is estimated to affect up to two billion people worldwide and can result in cell-mediated immune dysfunction and cognitive impairment [19]. Many epidemiological studies have shown a relationship between zinc content in the diet and cancer risk [20,21].

In cancer patients, deficiencies of micronutrients such as selenium or zinc can already be present at the time of diagnosis [22,23]. For zinc deficiency, the results of previous studies are conflicting [21,24,25]. Selenium deficiency at the time of diagnosis, however, is associated with worse overall survival rates [22,26,27]. Selenium status can further decrease during radiotherapy, especially after prior chemotherapy [28,29]. Low selenium levels have been shown in breast, colorectal, and pancreatic cancer patients [30,31,32,33]. 

The aim of this observational study was to determine the prevalence of selenium and zinc deficiency in patients with breast, colon, and pancreatic cancer at the beginning of rehabilitation, and to determine the effect of routine selenium supplementation on selenium status at the end of rehabilitation and three months after rehabilitation in a cancer rehabilitation center in Germany. In addition, global routine health-related quality of life (HRQOL) was analyzed at the beginning and the end of rehabilitation, as well as three months after.

## 2. Materials and Methods

### 2.1. Study Design

An ethics committee vote was not necessary, as only clinic intern register data were used for this retrospective cross-sectional study. The data were routinely collected in patient care. Written informed consents for the data analysis of patient data were obtained from all participating patients in accordance with the Declaration of Helsinki. The clinical data were recorded in a clinic specialized in cancer rehabilitation (Inselsberg Klinik Bad Tabarz, Germany). The data of patients treated for breast cancer, pancreatic cancer, or colon cancer, in which selenium and zinc status were determined as part of their treatment, were anonymized in a period from 2016 to 2022. The anonymized data included age, sex, diagnosis, therapy, prior supplementation, and selenium or zinc supplementation. Selenium and zinc concentrations in whole blood were measured at three time points: admission, discharge (after three weeks), and three months after discharge. 

### 2.2. Measurement of Whole Blood Selenium and Zinc

Whole blood selenium samples were obtained at the beginning and the end of the rehabilitation stay at the Inselsberg Clinic Bad Tabarz, as well as after three months, using tubes for trace elements/metal analytic. Blood samples were sent to a certified laboratory (biosyn Arzneimittel GmbH, Fellbach, Germany). Selenium and zinc levels were measured by microwave digestion and flameless atomic absorption spectrometry, according to the method of Winnefeld et al. [34]. Selenium status in whole blood <100 µg/L was defined as deficient, using the reference range defined by German authorities [34]. The reference range for zinc was 4.0–7.5 mg/L. 

Selenium was supplemented in the form of sodium selenite (tablets containing 300 µg selenium, selenase, biosyn Arzneimittel GmbH, Fellbach) for three weeks during rehabilitation and three months thereafter. At the beginning of the analyzed time in 2016, 300 µg selenium per day was supplemented. From 2017 to 2022, the daily selenium supplementation was increased to 600 µg selenium.

### 2.3. European Organization for Research and Treatment of Cancer (EORTC) Quality of Life Questionnaire Core-30 (EORTC QLQ-C30)

Data from routine HRQOL at an inpatient cancer rehabilitation center were analyzed. Patients completed the European Organization for Research and Treatment of Cancer (EORTC) Quality of Life Questionnaire Core-30 (EORTC QLQ-C30) at the beginning, the end, and three months after the multidisciplinary rehabilitation treatment. Missing values in filled out questionnaires were 1.5%. All included patients completed a EORTC QLQ-30 questionnaire at the beginning of rehabilitation (Pre-Rehab). At discharge (Post-Rehab), in total, five EORTC QLQ-30 questionnaires were missing, all from pancreatic cancer patients. The return rate for EORTC QLQ-30 questionnaires three months after rehabilitation was 35% (27% for breast cancer; 37% for colon cancer; 36% for pancreatic cancer).

### 2.4. Statistical Analysis

All data were stored and analyzed using GraphPad 9.1. All continuous data are presented as means ± standard deviation (SD), and the differences were assessed by one-way analysis of variance (normal distribution) or Kruskal–Wallis H test (non-normal distribution). All categorical data are presented as percentages; the differences were assessed by Pearson’s chi-squared test. Differences between whole blood selenium concentrations in continuous variables were analyzed by Student’s *t*-test (normal distribution) and the Mann–Whitney test (non-normal distribution) for independent samples. One-way ANOVA was used to compare three or more groups (*p* trend). The size of change at admission, discharge, and three months after discharge were evaluated using Cohens’ d. Effect sizes (ES) were considered as small (d = 0.2), medium (d = 0.5), or large (d = 0.8) [35]. All *p* values were two-sided statistical tests and were considered statistically significant if <0.05.

## 3. Results

### 3.1. Patient Characteristics

The study included 271 cancer patients in rehabilitation. Age, sex, and therapy varied depending on tumor location (Table 1). In pancreatic cancer patients, baseline selenium status was 81.6 ± 14.6 µg/l in women and 79.5 ± 16.6 µg/L in men (*p* = 0.8252). The results were comparable in colon cancer patients, with 89.1 ± 18.1 µg/L in women and 90.3 ± 16.9 µg/L in men (*p* = 0.8617). As breast cancer patients were all female and displayed significantly higher selenium levels, they were excluded from an overall analysis. Overall analysis with pancreatic and colon cancer patients showed no significant difference in selenium status between women and men (86.0 ± 17.0 µg/L vs. 85.6 ± 17.5 µg/L; *p* = 0.9453). In breast and pancreatic cancer patients, age was not associated with selenium status (*p* = 0.9187 and *p* = 0.8022). In contrast, baseline selenium levels decreased with age in colon cancer patients (*p* < 0.0001).

Zinc status was not associated with age. In colon cancer patients, zinc levels were not associated with sex (*p* = 0.7225). In contrast, zinc status was associated with sex in pancreatic cancer patients (*p* = 0.0008). Breast cancer patients were included in overall analysis as their zinc levels were comparable. Overall analysis showed a significant association between zinc status and sex (*p* = 0.0075). Selenium and zinc status was not associated with tumor size, grading, chemotherapy or radiotherapy.

Prior supplementation of selenium or zinc was documented. Only two patients supplemented zinc before rehabilitation. Prior selenium supplementation was rare in pancreatic and colon cancer patients. In contrast, more than every fourth breast cancer patient supplemented selenium. Analysis showed that there was no significant difference in baseline selenium status if breast cancer patients supplemented selenium prior to rehabilitation (*p* = 0.2137). Therefore, patients with prior selenium supplementation were included in the analysis. 

### 3.2. High Prevalence of Selenium Deficiency Depending on Tumor Localization

While selenium deficiency was prevalent with high incidence in cancer patients in rehabilitation (73%), zinc deficiency was rare (1%). Selenium deficiency was associated with tumor localization (Table 2). Prevalence of selenium deficiency was 90% in patients with pancreatic cancer, 74% in patients with colon cancer, and 36% in breast cancer patients. In total, 13 of 50 breast cancer patients supplemented various dosages of selenium before rehabilitation. Selenium deficiency was less prevalent in this group (23% vs. 41%), but the difference was not significant (*p* = 0.2591).

### 3.3. Selenium Supplementation during Rehabilitation Corrects Selenium Deficiency

Selenium supplementation during rehabilitation significantly improved the selenium status in all patients (*p* < 0.0001). Initially, breast and colon cancer patients were supplemented with 300 µg selenium per day. Then, supplementation was increased to 600 µg selenium per day. Due to their low selenium status, all pancreatic cancer patients were supplemented with 600 µg selenium per day from the beginning.

**Figure 1:**
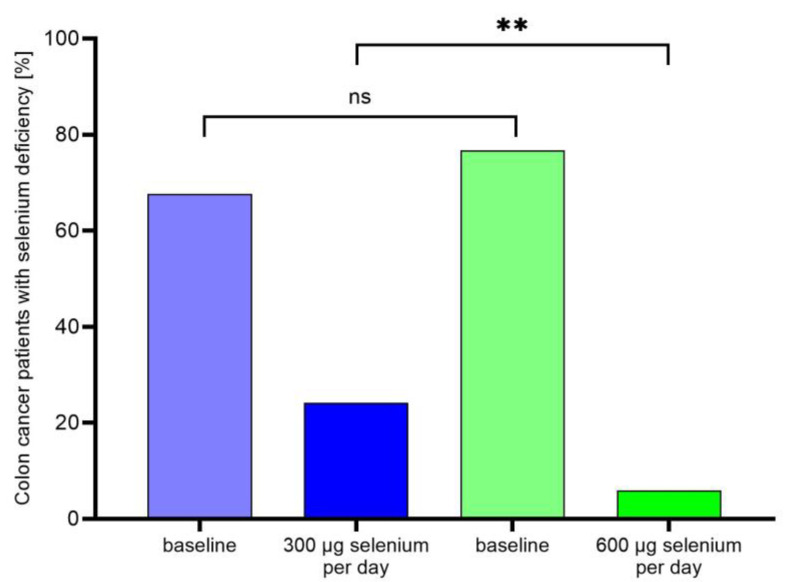
Selenium deficiency in colon cancer patients after supplementation with 300 µg or 600 µg selenium per day. ** *p* < 0.01; n.s. not significant.

There was no significant difference in selenium status at admission between patients with colon cancer receiving 300 µg or 600 µg selenium per day (Figure 1). At the end of rehabilitation, selenium status was significantly higher in breast and colon cancer patients who received 600 µg selenium per day compared to patients only receiving 300 µg (breast cancer: 141.2 ± 17.1 µg/L vs.127.1 ± 18.8 µg/L, *p* = 0.0076; colon cancer 126.2 ± 17.5 µg/L vs. 116.5 ± 18.6 µg/L, *p* = 0.0239). In colon cancer patients, the proportion of patients with selenium deficiency was significantly lower at the end of rehabilitation when receiving 600 µg selenium per day (5.9% vs. 24.2%; *p* = 0.0042) (Figure 1).

### 3.4. Different Development of Whole Blood Selenium Concentration

**Figure 2:**
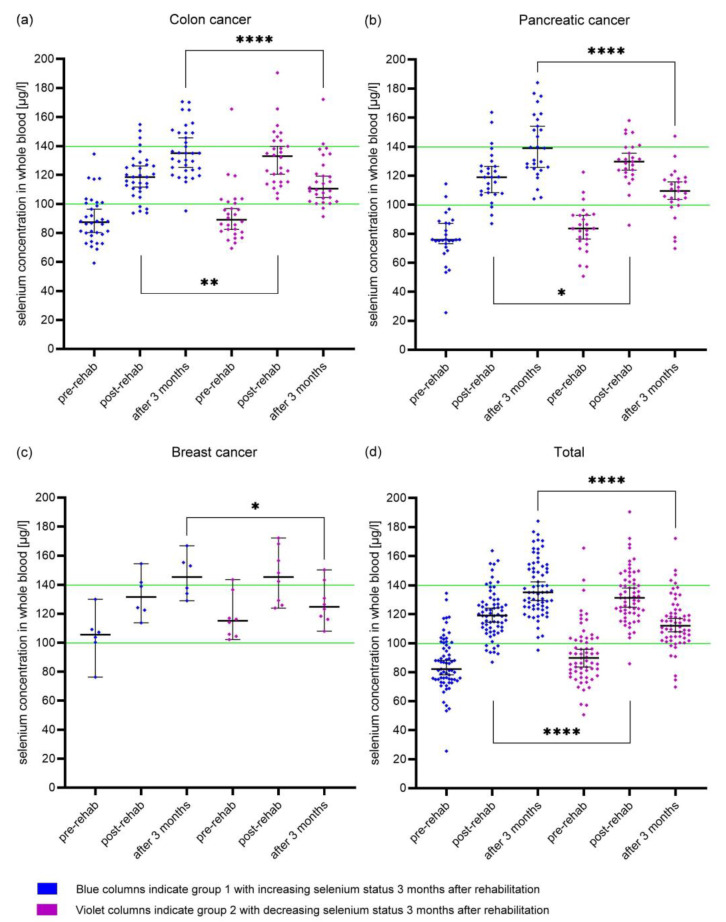
Median whole blood selenium concentration (95% CI) with individual values in (**a**) colon (blue *n* = 34; red *n* = 28), (**b**) pancreas (blue *n* = 27; red *n* = 25), (**c**) breast cancer patients (blue *n* = 6; red *n* = 8), and (**d**) all cancer patients with three measurements. Blue values: increasing selenium status 3 months after rehabilitation; violet values: decreasing selenium status 3 months after rehabilitation. Green lines indicate the German reference range for whole blood selenium concentration. * *p* < 0.05; ** *p* < 0.01; **** *p* < 0.0001.

When looking at the individual values of the cancer patients, from whom three measured values were available, there were two different groups. In the first group, the selenium status continued to increase three months after rehabilitation or remained on a comparable level. In the second group, the selenium status decreased three months after rehabilitation. This different development in selenium status was significant in all three tumor types, while the selenium values at the beginning of rehabilitation (pre-rehab) were not significantly different (Figure 2). The values at the end of rehabilitation (post-rehab) in the second group were significantly higher for colon and pancreas cancer patients. 

Mean age values were comparable in both groups (67.8 ± 11.2 vs. 66.8 ± 11.7). In addition, ongoing chemotherapy during rehabilitation as well as planned chemotherapy thereafter were not associated with selenium status development. A decrease in selenium status three months after rehabilitation occurred in cancer patients with and without selenium supplementation before rehabilitation.

### 3.5. Differences in EORTC QLQ-C30 Results Depending on Tumor Localization

Overall, rehabilitation improved functional scales and quality of life (Table 3, Table 4 and Table 5). Baseline values were worst in pancreatic cancer patients and best in breast cancer patients.

The four key domains of the EORTC QLQ-C30—physical functioning, emotional functioning, fatigue, and pain—all improved significantly during rehabilitation (Table 3, Table 4 and Table 5). Assuming a change of ≥10 points for a clinically significant improvement, this was only partially achieved in the four key domains and global quality of life during rehabilitation. 

Global quality of life improved more than 10 points during rehabilitation in breast, colon, and pancreatic cancer. However, global quality of life values decreased 1.5 to 4.3 points in all three cancer types. The effect size was large at the end of rehabilitation in breast cancer patients (d = 0.820) but was only moderate three months after discharge (d = 0.535). In pancreatic cancer patients, the effect size was only barely moderate (*p* = 0.511) during rehabilitation. Effect size further decreased after rehabilitation until only a small effect remained (d = 0.389). In colon cancer patients, the effect size of global quality of life was moderate during and after rehabilitation (d = 0.632 and d = 0.516, respectively)

In breast cancer, the key domains of fatigue and pain improved 10.4 points and 10.9 points, respectively, during rehabilitation. A mean value better than the thresholds for clinical importance, as defined by Giesinger et al., was only achieved for fatigue (value below 39 points) [36]. However, both values were worse three months after discharge. The key domain, physical functioning, still improved three months after discharge, with a medium effect size (d = 0.588). Emotional functioning improved 17.6 points during rehabilitation. Three months after discharge, mean value decreased 17.1 points, displaying no effect (d = 0.027) compared to a medium effect size at discharge (d = 0.739). Global quality of life was the only value which showed a large effect size (d = 0.820) at the end of rehabilitation. 

In colon cancer, the mean values in physical functioning and emotional functioning showed a development comparable to that in breast cancer (Table 4). Physical functioning improved 9.9 points three months after discharge. Emotional functioning improved 11.5 points during rehabilitation and declined 7.9 points three months after discharge. Fatigue improved 12.9 points during rehabilitation. Mean value worsened slightly but stayed below the threshold of 39 points [36]. In contrast to breast cancer, mean values for pain improved until three months after discharge, with 16.2 points, which is below the threshold of 25 points, and showed a medium effect size (d = 0.518) [36]. In colon cancer, the symptom of diarrhea improved significantly by decreasing 10.3 points during rehabilitation. However, the mean value increased 6.3 points again at three months after discharge. Global quality of life was the only value which showed a medium effect size (d = 0.632) at the end of rehabilitation and three months after discharge (d = 0.516).

In pancreatic cancer, the key domain of fatigue improved 11.8 points during rehabilitation and remained stable until three months after discharge. The mean values stayed far above the threshold of 39 [36]. Mean values for pain improved until three months after discharge (10.1 points) and remained above the threshold of 25 [36]. Physical and emotional functioning improved 8.9 and 9.3 points, respectively, during rehabilitation. The mean value remained almost 20 points below the threshold of 83 for physical functioning [36]. In pancreatic cancer, appetite loss was a major burden compared to in breast or colon cancer (47.3 vs. 9.8 vs. 18.3). Appetite loss improved 20.4 points until three months after discharge with a medium effect size (d = 0.541). Also, constipation and diarrhea displayed significant changes during rehabilitation and three months after discharge. While diarrhea improved 15.9 points during rehabilitation, constipation worsened 19.9 points in the same time frame. In contrast, constipation was improved by 28.3 points three months after discharge, while diarrhea worsened 12.8 points.

### 3.6. Differences in EORTC QLQ-C30 Results Depending on Selenium Status

The four key domains of the EORTC QLQ-C30 and global quality of life were further analyzed in colon and pancreas cancer patients, regarding differences between the two selenium status development groups. The sample size for breast cancer patients was too small for this sub-analysis (*n* = 14).

**Figure 3:**
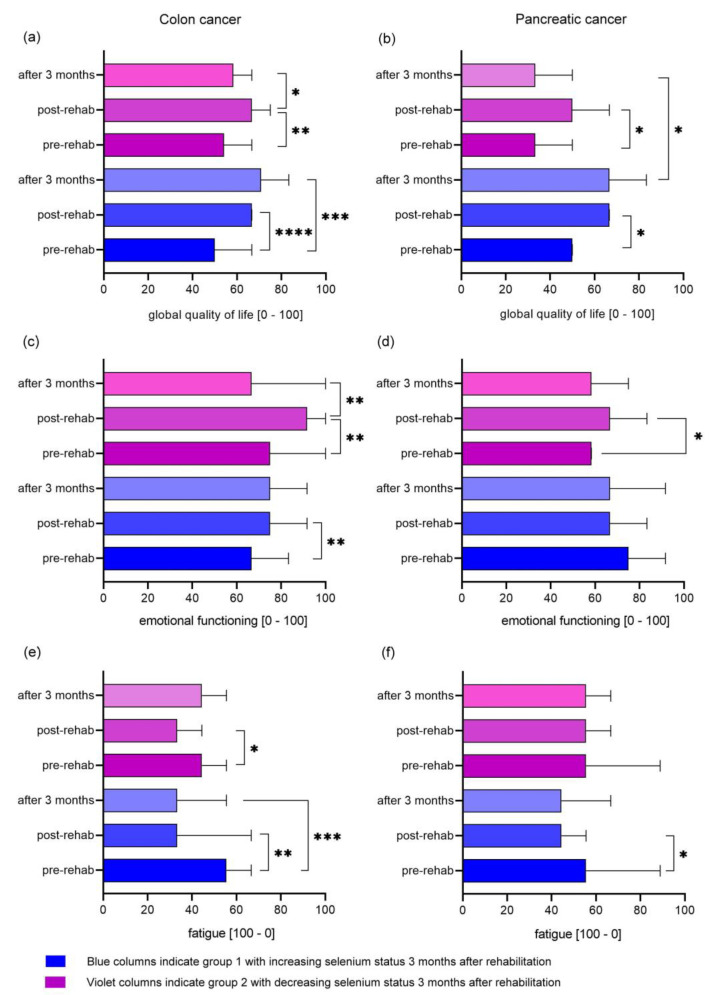
Median EORTC QLQ-C30 values (95 % CI) at the beginning of rehabilitation (pre-rehab), at the end of rehabilitation (post-rehab), and after 3 months. (**a**) + (**c**) + (**e**) colon cancer patients; (**b**) + (**d**) + (**f**) pancreatic cancer patients. Blue values: increasing selenium status 3 months after rehabilitation; violet values: decreasing selenium status 3 months after rehabilitation. Scale from 0 to 100 with 100 reflecting the best possible score for functioning scales and the worst score for symptom scales [37]. * *p* < 0.05; ** *p* < 0.01; *** *p* < 0.001; **** *p* < 0.0001.

In colon cancer patients, global quality of life was significantly higher in group 1 at discharge (*p* < 0.0001) and three months after rehabilitation compared to the beginning of rehabilitation (*p* = 0.0002) (Figure 3a). In contrast, while initially, global quality of life significantly increased at the end of rehabilitation in group 2 (*p* = 0.0039), it decreased again significantly until three months after rehabilitation (*p* = 0.0134). At three months, no significant difference was observed compared to the start of rehabilitation in group 2. The improvement of global quality of life during rehabilitation was also significant in pancreatic cancer patients in both groups (Figure 3b). As in colon cancer, after three months, scores decreased in the second group. In addition, global quality of life was significantly better in group 1, with increasing selenium status three months after rehabilitation compared to group 2, with decreasing selenium levels (Figure 3b).

In pancreatic cancer, values for physical functioning were significantly higher in group 1 three months after rehabilitation compared to group 2 (*p* = 0.0245). At the beginning of rehabilitation, values for physical functioning were not significantly different. Developments in physical functioning were comparable between both groups.

Emotional functioning values worsened significantly in group 2 of colon cancer patients while remaining stable in group 1 (*p* = 0.0059) three months after rehabilitation (Figure 3c). Values for emotional functioning in group 2 also decreased in pancreatic cancer patients after three months. However, the change was not significant (Figure 3d).

Scores for fatigue improved significantly at the end of rehabilitation and three months after discharge in group 1 of colon cancer patients (Figure 3e). In contrast, fatigue significantly improved only during rehabilitation in group 2 and returned to almost baseline three months after rehabilitation. In pancreatic cancer, only the first group significantly improved fatigue scores during rehabilitation, while there was no change in group 2 (Figure 3f).

In colon cancer patients, pain scores improved significantly at the end of rehabilitation and three months after in group 1 (*p* = 0.0005 and *p* = 0.0413, respectively). In group 2, there was no significant change in pain scores. In contrast, in pancreatic cancer, pain scores significantly decreased at the end of rehabilitation and three months after in group 2 (*p* = 0.0176 and *p* = 0.0349, respectively). In group 1, with increasing selenium values, there was no significant change in pain scores.

## 4. Discussion

Cancer patients beginning rehabilitation display a high prevalence of selenium deficiency depending on tumor localization (pancreas > colon > breast). In contrast, zinc deficiency was rare. Daily selenium supplementation of 600 µg was more efficient to correct selenium deficiency compared to 300 µg selenium per day. Rehabilitation and increasing selenium status were associated with improved global quality of life, physical and emotional functioning, and fatigue.

### 4.1. Selenium Deficiency in Cancer Patients in Rehabilitation

Selenium deficiency was common in cancer patients at the beginning of rehabilitation in a German rehabilitation clinic. In contrast, zinc deficiency was rare. With 90%, prevalence of selenium deficiency was highest in pancreatic cancer patients. In colon cancer, prevalence of selenium deficiency was also high, with 73%, while breast cancer patients were only deficient in 36% of cases. 

To our knowledge, this is the first analysis of micronutrient status of cancer patients in rehabilitation. So far, selenium and zinc levels were determined at the time of cancer diagnosis [22,23]. Selenium levels were found to be lower in cancers of the pancreas, gastric tract, and breast, compared to cancer of prostate, throat, or lung [30,31,38,39,40]. This is in accordance with the results for colon and pancreatic cancer in this study. Interestingly, the selenium levels in breast cancer patients were higher, but while the prevalence was much lower, one-third of the patients were still selenium deficient. 

Selenium supplementation before rehabilitation was more frequent in breast cancer patients compared with colon cancer and pancreatic cancer patients (28%, 8%, and 1%, respectively). Selenium deficiency was less prevalent in breast cancer patients, supplementing selenium before rehabilitation (23% vs. 41%), but the difference was not significant. An online survey with breast cancer patients in Germany showed that selenium was the most frequent used complementary therapy with more than 50% [41]. In an Italian multicenter survey, breast cancer patients were more likely to use complementary therapy compared to patients with colon or pancreatic cancer [42]. In addition, disease severity could have been lower in breast cancer patients, as disease severity has been negatively associated with selenium levels [27,33,43]. Radiotherapy can also cause a decrease in selenium status, especially with prior chemotherapy [28,29]. However, three-quarters of the breast cancer patients had received radiotherapy and one-third had also received chemotherapy before rehabilitation.

### 4.2. Selenium Supplementation Corrects Selenium Deficiency

Selenium supplementation was a standard of care in the rehabilitation center during the analyzed period. At the beginning, breast and colon cancer patients were supplemented with 300 µg selenium per day. Follow-up measurements at the end of rehabilitation indicated that the selenium dosage was not sufficient to reliably correct selenium deficiency in those cancer patients. Therefore, selenium supplementation was increased to 600 µg selenium per day. Due to their low selenium status, all pancreatic cancer patients were supplemented with 600 µg selenium per day from the beginning.

Retrospective analysis in this study confirmed that a selenium supplementation with 600 µg per day decreased the proportion of patients with selenium deficiency more efficiently compared to 300 µg selenium. Only 6% were still selenium deficient with the higher selenium dosage compared to almost 25% when receiving 300 µg selenium per day.

Interestingly, when looking at the selenium status in each patient after supplementation, they were divided into two groups irrespective of tumor localization. At the beginning of rehabilitation, selenium status was comparable. The first group showed an increasing selenium concentration in whole blood during selenium supplementation at the end of rehabilitation and three months after. In the second group, selenium status was significantly higher post-rehabilitation but then markedly decreased three months after rehabilitation compared to the first group.

Differences in the discharge-to-three-month follow-up period might be a reason for this different development in selenium status. Selenium supplementation was a standard of care during rehabilitation, and further selenium supplementation was recommended to the resident physician. Recommended selenium dosage depended on the selenium status of the patient. The target value was 150 µg/L selenium in whole blood. The recommendation for cancer patients with selenium deficiency (<100 µg/L) was 600 µg selenium per day and 300 µg selenium per day for everyone else.

However, selenium dosages above 70 µg per day need a prescription in Germany. Thus, ongoing selenium supplementation depended on many different physicians and might have occurred at lower dosages or been discontinued after discharge. In addition, statutory health insurances pay for selenium supplementation, in the form of prescription drugs, only if selenium deficiency is proven beforehand. Physicians might have tended to use private prescriptions. Therefore, for some patients, the additional financial burden might have affected adherence to continuous supplementation.

Another aspect that should be considered regarding the observed differences in the development of selenium levels in these two groups is a changed selenium requirement after rehabilitation. A considerable proportion of the cancer patients had planned chemotherapy after rehabilitation, which might decrease selenium values [28,29]. However, ongoing chemotherapy during rehabilitation and planned chemotherapy thereafter were not associated with selenium status development. 

### 4.3. Effect of Rehabilitation and Selenium Supplementation on Quality of Life

Overall, global quality of life improved during rehabilitation more than 10 points in all three cancer types. Snyder et al. showed that a 10-point EORTC-QLQC30 score change represents changes in supportive care needs [44]. This indicates that rehabilitation equally improved quality of life in breast, colon, and pancreatic cancer. The long-term effect was determined measuring EORTC-QLQC30 scores again three months after rehabilitation. 

Global quality of life values decreased between two and four points. This result is in accordance with the data from Lamprecht et al., who also measured global quality of life at the beginning, the end, and three months after rehabilitation [45]. Starting values for global quality of life values were comparable, and development was almost identical in breast and colon cancer patients. In contrast, mean values of global quality of life was more than 10 points lower in pancreatic cancer patients compared to EORTC QLQ-C30 reference values for metastatic pancreatic cancer [46]. On the other hand, the development of global quality of life was comparable to breast and colon cancer patients. This is in contrast to the results of Singer et al. [47]. EORTC-QLQC30 scores were assessed at admission and four months after rehabilitation in cancer patients older than 69 years. Global quality of life improved more than 25 points for gastrointestinal tumors, while in breast cancer patients, the improvement was only in the range of 5 points [47]. 

Interestingly, global quality of life values developed differently three months after rehabilitation depending on selenium status in pancreatic and colon cancer. Global quality of life only decreased in the group of cancer patients, which displayed decreasing selenium values after rehabilitation. In pancreatic cancer, global quality of life was significantly lower in this group compared to the group with increasing or stable selenium status. To our knowledge, this is the first description of a clinically significant effect of selenium status on global quality of life in cancer patients. In elderly healthy volunteers, selenium supplementation did not appear to benefit mood or quality of life [48].

### 4.4. Effect of Rehabilitation and Selenium Supplementation on Key Domains

The long-term effects of rehabilitation varied in the key domains of physical and emotional function, fatigue, and pain. Colon cancer patients estimated their physical functioning at the start of rehabilitation more poorly than breast cancer patients, which is in line with previous results [45]. In contrast, EORTC QLQ-C30 reference value for physical functioning in metastatic pancreatic cancer was more than 20 points better compared to the starting value in this study (78.2 vs. 54.5 points) [46]. Physical functioning improved further or remained stable in the three months after rehabilitation. As for global quality of life, values for physical functioning displayed comparable starting values and development for breast and colon cancer patients as Lamprecht et al. [45]. The change of approximately 10 points was also consistent with previous results [47]. Pancreatic cancer patients displayed lower physical functioning values three months after rehabilitation, when their selenium status decreased in this timeframe.

Breast and pancreatic cancer patients at the start of rehabilitation and three months thereafter rated emotional functioning significantly more poorly compared to colon cancer patients. Lamprecht et al. also showed this in breast cancer [45]. In contrast, emotional functioning value was more than 10 points lower in this earlier study. Results for emotional functioning are conflicting, as another study had lower initial value in gastrointestinal cancer compared to breast cancer [47]. Emotional functioning significantly improved during rehabilitation, especially in breast and colon cancer patients. However, this improvement did not last. While this was already shown before [49], in previous trials there was still a significant improvement [45,47]. Here, initial emotional functioning values were not significantly different compared to those three months after rehabilitation. When taking only those patients with all three selenium status measurements into consideration, improvement of emotional functioning remained in colon cancer patients with increasing or stable selenium levels after rehabilitation. 

During rehabilitation, fatigue scores improved most compared to all other symptom scales. While this applied for all tumor types, the initial fatigue score was significantly worse in pancreatic cancer patients. It was also 15 points higher compared to the EORTC QLQ-C30 reference value in metastatic pancreatic cancer [46]. Fatigue scores worsened again three months after the end of rehabilitation but were still significantly better compared to initial values. Similar results were shown by Lamprecht et al., while fatigue score was worse four months after rehabilitation in breast cancer in another trial [45,47]. In colon cancer patients with increasing or stable selenium levels after rehabilitation, the positive effect of rehabilitation on fatigue persisted longer compared to those with decreasing selenium status.

A possible positive effect of selenium, especially on fatigue, was shown in a clinical trial with children and adolescents with cancer [50]. After one year of supplementation with selenium, fatigue scores decreased in patients with solid tumors and hematological cancer [50]. In a recent trial, selenium biomarkers, such as plasma selenium, glutathione peroxidase, and selenoprotein P, showed linear correlations in patients with chronic fatigue syndrome without reaching saturation, indicative of Se deficiency [51].

As colorectal cancer survivors are more likely to report fatigue [52,53] and often display selenium deficiency, the results indicate that they could benefit when selenium status is assessed and selenium deficiency corrected. A long-term selenium supplementation seems to be necessary to maintain an adequate selenium status in cancer patients so the positive effects of rehabilitation persist longer. The symptom burden and prevalence of selenium deficiency was highest in pancreatic cancer patients in this trial. Therefore, the conclusion for colorectal cancer patients could also apply to patients with pancreatic cancer.

### 4.5. Strengths and Limitations

This study has a number of limitations, including a retrospective single-center design, inherent heterogeneity of cancer patients, lack of a control group (patients not undergoing rehabilitation) and low number of patient data three months after discharge regarding selenium and zinc status, and returned EORTC QLQ-30 forms. Another important factor could have been the refusal of further prescription of selenium medication, as selenium dosages over 70 µg per day are prescription only in Germany. After rehabilitation, most often the family doctor takes over further treatment and necessary prescriptions. Therefore, the patient is dependent on the attending physician. The results of our study imply that there might be reservations in Germany to treat selenium deficiency continuously. 

The strength of this study is the inclusion of three different cancer types and the numbers of patients in rehabilitation who received comparable treatment, including assessment of selenium and zinc status and selenium supplementation as a standard of care.

## 5. Conclusions

Selenium deficiency is common in cancer patients starting rehabilitation. Prevalence of selenium deficiency ranged between 36 and 90% depending on tumor location. Selenium supplementation with 600 µg was highly efficient to correct selenium deficiency. In cancer patients with decreasing selenium status three months after rehabilitation, values of global quality of life, physical and emotional functioning, and fatigue were back to the values at the beginning of rehabilitation. This indicates that the positive effects of rehabilitation persisted longer when selenium status did not decrease after rehabilitation.

## Figures and Tables

**Table 1:** Descriptive characteristics of patients.

Characteristic	Breast Cancer	Pancreatic Cancer	Colon Cancer
Sample, *n*	50	101	120
Sex (f/m)	98%/2%	64%/36%	53%/47%
Age, years, mean ± SD	62 ± 10	71 ± 11	69 ± 11
Operation	100%	87%	97%
Chemotherapy			
planned	26%	40%	7%
ongoing	0%	20%	6%
after	34%	28%	41%
none	40%	11%	47%
Radiotherapy	76%	4%	21%
Hormone therapy	78%	-	-
Selenium status ^†^, µg/L, mean± SD	107.2 ± 18.4	80.5 ± 15.5	90.0 ± 17.6
Prior selenium supplementation	28%	1%	8%
Zinc status ^§^, mg/L, mean ± SD	5.9 ± 0.8	6.0 ± 0.8	6.0 ± 0.9
Prior zinc supplementation	0%	0%	2%

^†^ Selenium concentration in whole blood; ^§^ zinc concentration in whole blood.

**Table 2:** Selenium and zinc deficiency in cancer patients.

Characteristic	Breast Cancer	Pancreatic Cancer	Colon Cancer
Sample, *n*	50	101	120
Selenium deficiency ^†^			
beginning of rehabilitation	36%	90%	74%
end of rehabilitation	4%	7%	11%
3 months after rehabilitation	0%	6%	3%
Zinc deficiency ^§^			
beginning of rehabilitation	0%	0%	1%
end of rehabilitation	0%	0%	1%
3 months after rehabilitation	0%	0%	1%

^†^ Selenium concentration in whole blood; ^§^ zinc concentration in whole blood.

**Table 3:** EORTC QLQ-30 mean scores (SD) of breast cancer patients at beginning of rehabilitation, end of rehabilitation, and three months thereafter.

	Pre-Rehab	Post-Rehab	3 Months after Rehabilitation	p-Trend
Functional scales and global QoL				
Physical functioning	73.6 (16.5)	80.0 (16.7)	83.1 (14.6)	0.0012
Role functioning	56.1 (26.5)	68.7 (26.1)	62.8 (26.5)	0.0253
Emotional functioning	57.6 (27.3)	75.4 (20.3)	58.3 (21.0)	<0.0001
Cognitive functioning	67.7 (29.5)	76.9 (24.5)	61.6 (26.7)	0.0752
Social functioning	64.2 (27.5)	74.8 (24.1)	74.4 (26.0)	0.0191
Global QoL	55.6 (15.9)	68.4 (15.3)	64.1 (15.7)	<0.0001
Symptom Scales				
Fatigue	48.6 (27.6)	38.2 (22.7)	45.3 (20.0)	0.0030
Nausea/Vomiting	5.5 (11.5)	2.7 (9.2)	2.6 (6.3)	0.0324
Pain	39.8 (32.2)	28.9 (27.4)	32.1 (26.8)	0.0329
Dyspnea	29.9 (33.9)	26.5 (28.9)	25.6 (20.0)	0.3564
Sleep disturbance	54.4 (31.7)	44.2 (36.3)	51.3 (37.6)	0.0395
Appetite loss	10.4 (24.0)	7.5 (20.7)	10.3 (16.0)	0.3470
Constipation	14.6 (25.6)	10.4 (21.9)	10.3 (28.5)	0.2214
Diarrhea	6.1 (16.2)	8.2 (21.0)	7.7 (14.6)	0.4660
Financial problems	19.1 (28.4)	15.0 (27.3)	7.7 (14.6)	0.1754

**Table 4:** EORTC QLQ-30 mean scores (SD) of pancreatic cancer patients at beginning of rehabilitation, end of rehabilitation, and three months thereafter.

	Pre-Rehab	Post-Rehab	3 Months after Rehabilitation	p-Trend
Functional scales and global QoL				
Physical functioning	54.5 (24.6)	63.4 (22.6)	63.2 (19.6)	<0.0001
Role functioning	41.5 (33.4)	54.5 (30.4)	52.9 (29.6)	0.0004
Emotional functioning	58.3 (28.5)	67.6 (25.6)	60.6 (28.1)	0.0041
Cognitive functioning	70.3 (27.4)	76.8 (23.6)	72.7 (26.2)	0.0653
Social functioning	51.7 (33.6)	62.0 (33.1)	57.9 (34.8)	0.0136
Global QoL	42.9 (23.1)	54.1 (20.8)	51.7 (21.3)	<0.0001
Symptom Scales				
Fatigue	60.9 (29.6)	49.1 (25.7)	49.4 (23.1)	<0.0001
Nausea/Vomiting	17.0 (26.2)	11.0 (20.4)	9.7 (13.4)	0.2027
Pain	41.1 (34.1)	32.3 (28.3)	31.0 (27.1)	0.0162
Dyspnea	31.6 (32.4)	31.9 (29.5)	27.8 (28.2)	0.4334
Sleep disturbance	44.6 (34.8)	36.9 (33.0)	38.9 (31.4)	0.0782
Appetite loss	47.3 (40.3)	37.6 (36.5)	26.9 (29.6)	0.0016
Constipation	17.7 (30.2)	37.6 (36.5)	9.3 (2.5)	<0.0001
Diarrhea	33.7 (35.8)	17.8 (30.2)	30.6 (33.2)	0.0007
Financial problems	18.9 (27.2)	16.9 (24.0)	24.1 (31.5)	0.3145

**Table 5:** EORTC QLQ-30 mean scores (SD) of colon cancer patients at beginning of rehabilitation, end of rehabilitation, and three months thereafter.

	Pre-Rehab	Post-Rehab	3 Months after Rehabilitation	p-Trend
Functional scales and global QoL				
Physical functioning	66.1 (21.5)	73.1 (20.4)	76.0 (19.3)	<0.0001
Role functioning	51.8 (33.4)	65.8 (28.2)	64.0 (27.8)	<0.0001
Emotional functioning	65.7 (24.5)	77.2 (22.6)	69.3 (27.7)	<0.0001
Cognitive functioning	81.7 (27.3)	82.7 (23.1)	72.0 (28.3)	0.0244
Social functioning	65.7 (31.8)	76.0 (27.4)	74.2 (28.0)	0.0257
Global QoL	51.9 (22.2)	64.4 (16.8)	62.9 (18.7)	<0.0001
Symptom Scales				
Fatigue	48.6 (27.6)	35.7 (26.1)	37.0 (27.1)	<0.0001
Nausea/Vomiting	6.4 (14.8)	5.4 (15.0)	4.2 (16.1)	0.2520
Pain	36.3 (32.9)	25.3 (27.5)	20.1 (26.1)	<0.0001
Dyspnea	23.8 (30.7)	26.8 (29.6)	16.7 (26.4)	0.0871
Sleep disturbance	43.5 (35.0)	38.0 (32.4)	34.9 (28.7)	0.0868
Appetite loss	18.3 (28.9)	12.3 (20.9)	11.4 (22.7)	0.2797
Constipation	17.1 (28.9)	11.1 (22.9)	12.4 (20.6)	0.3007
Diarrhea	27.7 (32.7)	16.4 (25.2)	22.7 (25.7)	0.0004
Financial problems	19.1 (29.3)	18.8 (30.3)	13.6 (28.1)	0.3350

## Data Availability

The data presented in this study are available on request from the corresponding author. The data are not publicly available due to privacy restrictions.
